# The effect of moral distress on emergency nurses' job burnout: the mediating roles of hospital ethical climate and moral resilience

**DOI:** 10.3389/fpubh.2025.1562209

**Published:** 2025-03-05

**Authors:** Shirong Wu, Yuqing Sun, Zhipeng Zhong, Huanmei Li, Banghan Ding, Qiuying Deng

**Affiliations:** ^1^The Second Clinical School of Medicine, Guangzhou University of Chinese Medicine, Guangzhou, China; ^2^The Second Affiliated Hospital of Guangzhou University of Chinese Medicine, Guangzhou, China

**Keywords:** emergency nurses, hospital ethical climate, job burnout, moral distress, moral resilience

## Abstract

**Background:**

Reducing nurse job burnout is vital for quality care and turnover reduction, particularly in emergency departments. Given that moral distress is a crucial predictor of job burnout, this study seeks to identify factors that can alter this relationship and its underlying mechanisms. The finding is essential for enhancing job satisfaction among emergency nurses and improving patient safety and healthcare quality.

**Methods:**

This study employed a cross-sectional design and was conducted in May 2024 among nurses in the emergency departments of five tertiary hospitals in Southern China. The survey instruments included the General Demographic Questionnaire, Moral Distress Scale-R (MDS-R), Hospital Ethical Climate Survey (HECS), Rushton Moral Resilience Scale (RMRS), and Maslach Burnout Inventory Human Services Survey (MBI-HSS). Descriptive analysis and Pearson correlation analysis were performed using SPSS 27.0. The structural equation model was constructed with AMOS 28.0 software, and Bootstrap testing was conducted.

**Results:**

The results showed that moral distress directly affected job burnout (β = 0.265, 95%CI [0.114, 0.391]). Hospital ethical climate and moral resilience both played mediating roles in the relationship between moral distress and job burnout (β = 0.161, 95%CI [0.091, 0.243]) (β = 0.216, 95%CI [0.123, 0.337]). Hospital ethical climate and moral resilience play chain mediating roles between moral distress and job burnout (β = 0.090, 95%CI [0.047, 0.161]).

**Conclusion:**

The hospital ethical climate and moral resilience play chain mediating roles between moral distress and job burnout. It is recommended that managers pay comprehensive attention to emergency nurses' moral distress. By improving the hospital ethical climate and enhancing nurses' moral resilience, the level of job burnout can be reduced.

## 1 Introduction

Job burnout (JB), a state of physical and mental exhaustion, is pervasive among nurses globally, particularly in high-stakes settings such as emergency departments (EDs) (1–3). In China, ED nurses face uniquely demanding challenges—including high workload, time pressure, and unpredictable critical incidents—that exacerbate burnout risk (4, 5). Left unaddressed, burnout not only threatens nurses' well-being and retention but also compromises healthcare quality and patient safety (6, 7). A critical yet understudied driver of burnout in this population is moral distress (MD), which occurs when nurses recognize the ethically appropriate action but cannot act due to internal or external constraints, leading to psychological imbalance (8). MD is endemic to emergency care due to its inherent ethical complexities, such as triage prioritization, end-of-life decisions, and resource allocation under time constraints (9). These dilemmas, compounded by the ED's unpredictable environment, impose sustained psychological burdens (10, 11), which studies link directly to burnout symptoms like emotional exhaustion and depersonalization (5, 12, 13). By elucidating this relationship, this study aims to identify actionable pathways to mitigate JB through targeted interventions.

**Figure 1:**
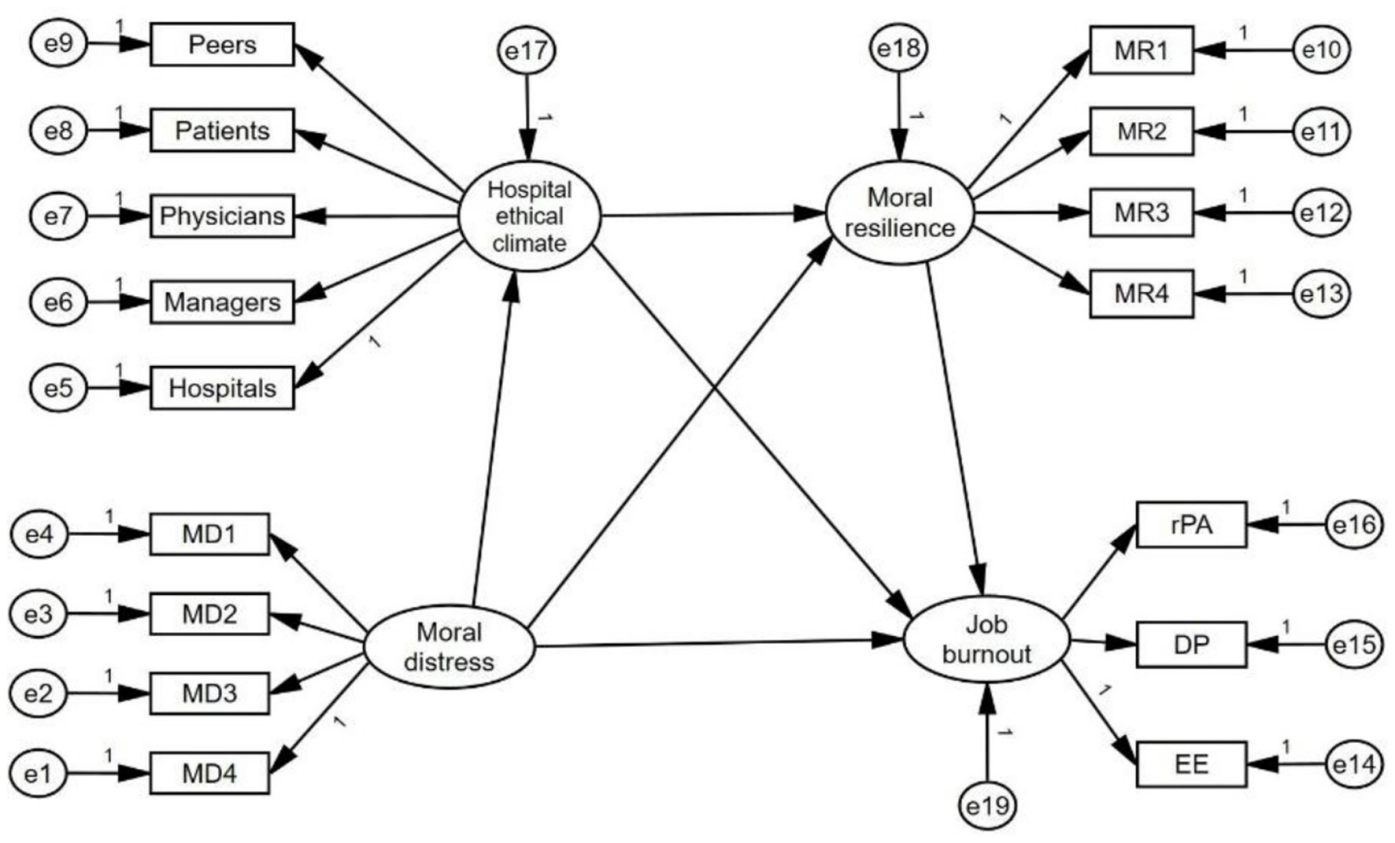
The theoretical model of this study. In this structural equation model (SEM), boxes represent manifest variables (directly observed or measured variables, such as survey items), while circles denote latent variables (unobserved constructs inferred from measured indicators, e.g., MD and HEC). Small circles with arrows pointing to boxes or circles represent residual variances, reflecting unexplained variance or measurement error in the model. One-way straight arrows (→) indicate hypothesized directional relationships between variables (e.g., MD → HEC → MR → JB).

Conservation of Resources (COR) theory posits that stress stems from the perceived loss or insufficiency of resources, categorized as internal resources (e.g., emotional resilience, self-efficacy, coping skills) and external resources (e.g., social networks, organizational support, access to tools) (14). In the context of emergency nurses' MD and JB, moral resilience (MR)—defined as the capacity to maintain moral integrity amid ethical challenges (15)—serves as a critical internal resource. It enables nurses to navigate moral complexity, reduce psychological distress (e.g., anxiety, depression) (16), and mitigate JB by preserving their sense of moral purpose (17). Empirical evidence suggests that higher MR effectively alleviate MD and JB (16). Thus, this study hypothesized: H1: MR mediates the relationship between MD and JB. Additionally, hospital ethical climate (HEC)—nurses' shared perceptions of how ethical issues are addressed in their workplace (18)—acts as an external resource. A positive HEC provides moral guidance and psychological support, reducing resource depletion caused by moral conflicts (19). Studies indicate that nurses perceiving a strong ethical climate report lower MD (19, 20) and JB (21). This suggests that HEC may attenuate the pathway from MD to JB by fostering an environment conducive to ethical decision-making. H2: HEC mediates the relationship between MD and JB. Furthermore, a positive HEC may enhance MR by offering institutional support that reinforces nurses' ethical confidence (22). This synergistic interaction between external (HEC) and internal (MR) resources might create a chain-mediating effect, wherein a supportive ethical climate bolsters MR, which in turn reduces JB risk (16, 19). H3: HEC and MR sequentially mediate the relationship between MD and JB (Figure 1).

Current research lacks empirical evidence on the pathways through which MD influences JB among emergency nurses. To address this gap, this study employs COR theory to examine the impact of MD on JB and further explores the chain-mediating roles of HEC (as an external resource) and MR (as an internal resource). By elucidating how these factors interact to mitigate JB, the findings aim to inform targeted interventions—such as fostering supportive ethical climates and enhancing MR training—to safeguard emergency nurses' well-being, reduce turnover, and ultimately improve healthcare quality and patient safety.

## 2 Methods

### 2.1 Design

This study employed a cross-sectional, online questionnaire survey design. The results were reported according to the Strengthening the Reporting of Observational Studies in Epidemiology (STROBE) guidelines (23).

### 2.2 Participants recruitment

This study used a convenience sampling method to recruit emergency department nurses from five tertiary hospitals in southern China in May 2024. The inclusion criteria for participants were: (1) holding a valid nurse practice license and having at least 1 year of clinical experience in the emergency department; (2) Providing informed consent and voluntarily participating in this study.

The exclusion criteria were: (1) nurses not engaged in clinical work (e.g., those in administrative roles); (2) Nurses absent from their posts due to maternity leave, sick leave, study leave, or other similar reasons.

According to Kendall's sample size estimation principle (24), the minimum sample size should be 10 times the number of research variables. This study involves 24 independent variables (8 demographic variables and 16 scale measurement variables), resulting in a baseline requirement of 240 valid samples. To account for potential invalid responses (estimated at 20%), the sample size was adjusted upward by 20% (240 × 1.2 = 288). Furthermore, considering the structural equation modeling (SEM) requirement that sample sizes should exceed 200 cases (24), the final determined minimum sample size for this study is 288 participants. Therefore, the actual sample collection should ensure a sample size >288.

### 2.3 Ethical considerations

The study was approved by the Ethics Committee of Guangdong Provincial Hospital of Chinese Medicine (No. YE2024-020-01, Guangzhou, Guangdong, China). Additionally, consent and support were obtained from the leaders of all participating hospitals. Data were collected via a questionnaire link on WeChat that directed participants to WenJuanXing platform (https://www.wjx.cn). The WeChat data center and WenJuanXing platform were employed to ensure that all data were used solely for research purposes and to provide robust data security measures, including data encryption, anonymization, and restricted access protocols, to protect participant confidentiality. All participants provided electronic informed consent and were informed that they could withdraw at any time without explanation or impact on their careers.

### 2.4 Data collection

Before the formal investigation, a pilot test was conducted with 20 nurses from the researcher's affiliated institution. This pre-experimental phase aimed to clarify any ambiguities regarding the questionnaire's questions, resolve them, and test the time to complete the survey. The participants from the pilot test were not included in the formal study.

The formal survey began with the corresponding author establishing collaborative relationships with the head nurses of the emergency departments in five hospitals to facilitate the dissemination of the study. Then, with the assistance of the head nurses, the research team set up separate WeChat groups for participants from each hospital, totaling 370 participants. The principal researcher introduced the study's purpose and precautions to each group. The survey link was distributed through the WeChat work groups, and the questionnaires were completed anonymously. The homepage of the questionnaire uses unified instructions, with each item set as a required answer, and only one questionnaire can be submitted from the same IP address. The survey lasted for 1 week. During this period, participants could contact the researchers via WeChat if they had any questions. As a token of appreciation, a small gift was prepared for each participant upon submission of the questionnaire.

The following criteria were used to exclude the questionnaires: (1) Completion Time: The average time to complete the questionnaire was between 3 and 15 min. To ensure the quality of the responses, questionnaires completed in <3 min were considered invalid. (2) Attention-Check Items: To ensure response validity, the questionnaire included instructional checks, which directly instructed participants to select a specific option (e.g., “Please select 'completely agree' to confirm you are reading carefully”). Responses failing to follow these instructions were excluded from analysis.

### 2.5 Instruments

#### 2.5.1 General demographic questionnaire

The research team designed it after a literature review, including gender, age, marital status, education level, years of working, professional title, and whether they participate in hospital or university ethics courses.

#### 2.5.2 MD scale

This study used the Chinese version of the Moral Distress Scale-Revised (MDS-R), which was translated and adapted by Sun et al. (25). The MDS-R was originally developed by Corley et al. and revised by Hamric to assess MD among nurses (26). The Chinese version of the MDS-R has demonstrated good reliability, with a Cronbach's alpha of 0.879 (25). The scale consists of 22 items across four dimensions: individual responsibility, not in the patient's best interest, value conflict, and harming the patient's interest. Each item included the frequency and intensity of nurses' MD, rated on a scale of 0 to 4. A score of 0 means never occurring or no distress, a score of 4 means very frequent or severe distress, and the product of the frequency and intensity scores is the score for each entry. The total scale score is the sum of all item scores, ranging from 0 to 352, with higher scores indicating more severe MD among nurses.

#### 2.5.3 HEC scale

This study used the Chinese version of the Hospital Ethical Climate Survey (HECS), which was translated and adapted by Wang in 2018, with a Cronbach's alpha of 0.915 (27). The HECS was originally developed by Olson (28) to measure clinical nurses' perceptions of the ethical climate in hospitals. The Chinese version of the scale consists of 25 items across 5 dimensions, focusing on the relationships between peers, patients, physicians, managers, and hospitals in the work environment. It utilizes a 5-point Likert scale, ranging from “Not at all” (1 point) to “Absolutely, yes” (5 points). The total score is calculated by summing the responses to all 25 items, with higher scores indicating a more positive perception of the HEC by nurses.

#### 2.5.4 MR scale

This study employed the Chinese version of the Rushton Moral Resilience Scale (RMRS), which was translated and adapted by YANG Q. et al. in 2022 (29). This version has shown good reliability, with a Cronbach's alpha of 0.763. The RMRS was originally developed by Heinze et al. to evaluate the level of MR among nurses (30). The Chinese version of the scale consists of 16 items in 4 dimensions of responses to moral adversity (4 items), moral efficacy (4 items), relational integrity (5 items), and personal integrity (3 items). It uses a 4-point Likert scale, ranging from 1 (“Disagree”) to 4 (“Agree”). The total score is calculated by summing the responses to all 16 items after reverse scoring the appropriate items, with possible scores ranging from 16 to 64. Higher scores indicate greater MR among nurses.

#### 2.5.5 JB scale

This study utilized the Chinese version of the Maslach Burnout Inventory Human Services Survey (MBI-HSS), which was translated and adapted by Feng et al. in 2004 (31). The original MBI-HSS was developed by Maslach to measure the level of JB among medical personnel (32). The Chinese version has demonstrated good reliability, with a Cronbach's α of 0.823. The scale consists of 22 items in 3 dimensions, including emotional exhaustion (9 items), deindividuation (5 items) and personal achievement (8 items). Likert 7-level scores were adopted, ranging from “never” to “every day”, with 0 to 6 points. All questions in the dimension of personal accomplishment were scored in reverse. The total score for each dimension is calculated by summing the responses to its respective items, with higher scores in emotional exhaustion and deindividuation indicating greater JB, while higher scores in personal accomplishment indicate lower JB.

### 2.6 Statistical analysis

The data were analyzed using IBM SPSS Statistics 27.0 for descriptive analysis of the demographic characteristics of the participants, and Pearson correlation analysis was employed to examine the relationships among the four variables. The study employed IBM SPSS Amos 28.0 Graphics to construct a mediation model with MD as the independent variable, HEC and MR as mediating variables, and JB as the dependent variable. The structural equation model (SEM) includes four latent variables and sixteen observed variables. The model fit was evaluated using the following indices: χ^2^/d*f* (Chi-square/Degrees of Freedom), GFI (Goodness-of-Fit Index), AGFI (Adjusted Goodness-of-Fit Index), NFI (Normed Fit Index), IFI (Incremental Fit Index), TLI (Tucker-Lewis Index), CFI (Comparative Fit Index), SRMR (Standardized Root Mean Square Residual), and RMSEA (Root Mean Square Error of Approximation). Detailed criteria for each index are provided in Table 1. The Bootstrap method was used to resample the data 5,000 times to test the mediating effect, with a confidence interval set at 95%. If the 95% confidence interval does not include 0, it indicates a mediating effect. All analyses were conducted using two-tailed tests, with a significance level of *a* = 0.05.

**Table 1:** Comparison of model fit for the modified and hypothetical models.

**Index**	***X*^2^/d*f***	**GFI**	**AGFI**	**NFI**	**IFI**	**TLI**	**CFI**	**SRMR**	**RMSEA**
Fitting criteria	1–3	>0.90	>0.90	>0.90	>0.90	>0.90	>0.90	>0.90	< 0.08
Fitted model	1.332	0.952	0.934	0.937	0.984	0.977	0.981	0.932	0.032

χ^2^*/df, Chi-square/Degrees of Freedom; GFI, Goodness-of-Fit Index; AGFI, Adjusted Goodness-of-Fit Index; NFI, Normed Fit Index; IFI, Incremental Fit Index; TLI, Tucker-Lewis Index; CFI, Comparative Fit Index; SRMR, Standardized Root Mean Square Residual; and RMSEA, Root Mean Square Error of Approximation*.

## 3 Results

### 3.1 Participant characteristics

During the formal study, a total of 365 questionnaires were collected from the WeChat groups that were initially established for 370 nurses. After the screening process, 42 questionnaires were excluded: 14 did not meet the required time criteria, and 28 failed the logical consistency test. This resulted in a final sample of 323 valid responses, with an effective response rate of 88.5%. This sample size exceeds the required minimum of 288.

The majority of the surveyed nurses were female (78.6%); the age distribution was most prevalent in the 20–30 and 30–40 age groups, accounting for 47.4% and 39.6%, respectively, 59.8% of the nurses were married, the majority held academic degrees (76.8%), and a significant proportion were senior nurses with extensive clinical experience (44.6%). Approximately half of the surveyed nurses had taken ethics courses in university (87.3%) and had received training in moral and ethical issues in the hospital (90.01%). Table 2 presents the demographic characteristics of the participants.

**Table 2:** Demographic characteristics of participants.

**Variable**	**Category**	**Frequency**	**Percentage (%)**
Gender	Male	69	21.4
	Female	254	78.6
Ages	20–30	153	47.4
	31–40	128	39.6
	>40	42	13.0
Marital Status	Single	133	40.2
	Married	190	59.8
Education Level	Below bachelor's degree	62	19.2
	Bachelor's degree	248	76.8
	Above bachelor's degree	13	4.0
Years of working	≤ 5	128	39.6
	6–10	87	26.9
	11–15	61	18.9
	>15	47	14.5
Professional Title	Nurse	62	19.2
	Senior nurse	144	44.6
	Supervisor nurse	101	31.3
	Deputy/Director nurse	16	5
Participation in hospital ethics courses	No	32	9.9
	Yes	291	90.1
Participation in University ethics course	No	41	12.7
	Yes	282	87.3

### 3.2 Normality test

Since maximum likelihood (ML) estimation was employed for parameter estimation in the SEM analysis, and ML requires the sample data to follow a normal distribution (33), a normality test was first conducted on the sample data. Generally, the criteria for passing the normality test are that the absolute values of skewness for all measurement items are <2, and the absolute values of kurtosis are <10 (34). Using SPSS 27.0 for data analysis, the maximum absolute values of skewness and kurtosis in the valid sample data were 0.983 and 0.876, respectively, both falling within the acceptable range. This indicates that the valid sample data conform to a normal distribution, allowing for subsequent confirmatory factor analysis.

### 3.3 Reliability analysis of the measurement model

In this study, the reliability of the questionnaire was assessed using Cronbach's alpha coefficient. Generally, a Cronbach's alpha coefficient >0.7 is considered acceptable, while values below 0.5 are deemed unsatisfactory (35). The Cronbach's alpha coefficients for the MDS-R, HECS, RMRS, and MBI-HSS were 0.792, 0.874, 0.929, and 0.966, respectively. These values indicate that the measurement model exhibits satisfactory reliability.

### 3.4 The scores of MD, HEC, MR, and JB

In this study, nurses reported a mean score of 51.05 ± 15.86 for MD and 90.64 ± 13.83 for HEC. The average score for MR was 40.14 ± 8.14. While the mean score for JB was 61.86 ± 24.19. Detailed scores for each dimension are shown in Supplementary Table S1.

### 3.5 Correlation analysis

The correlation analysis revealed a positive correlation between MD and JB (*r* = 0.612, *p* < 0.01). In contrast, MD was negatively correlated with both HEC (*r* = −0.481, *p* < 0.01) and MR (*r* = −0.274, *p* < 0.01). There was a negative correlation between MD and both HEC (*r* = −0.728, *p* < 0.01) and MR (*r* = −0.714, *p* < 0.01). Additionally, HEC was positively correlated with MR (*r* = 0.488, *p* < 0.01). Detailed results are shown in Supplementary Table S1.

### 3.6 Mediating effect analysis

**Figure 2:**
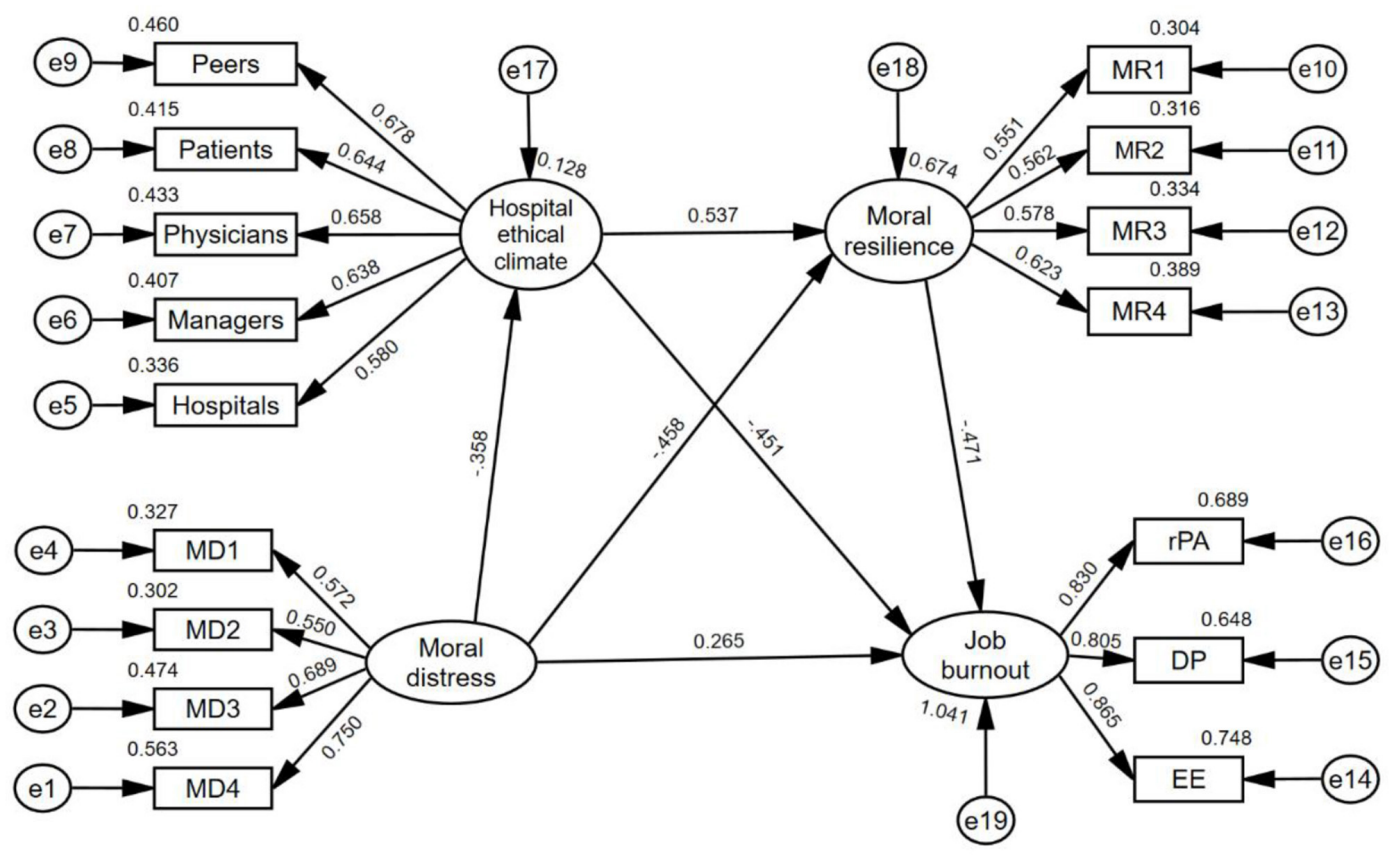
The mediating roles of hospital ethical climate and moral resilience between moral distress and job burnout. *MD1* individual responsibility; *MD2* not in the patient's best interest; *MD3* value conflict; *MD4* harming patient's interest; *MR1* responses to moral adversity; *MR2* moral efficacy; *MR3* relational integrity; *MR4* personal integrity; *EE* emotional exhaustion; *DP* depersonalization; *rPA* reduced personal accomplishment.

The hypothetical path model in Figure 1 was tested. The results showed that the model's overall fit was good (*X*^2^/d*f* = 1.332, GFI = 0.952, AGFI = 0.934, NFI = 0.937, GFI = 0.937, IFI = 0.984, TLI = 0.977, CFI = 0.981, SRMR = 0.932, RMSEA = 0.052), the specific fitting results are shown in Table 1, and the mediation model constructed is shown in Figure 2. The data on the intermediate path are standardized regression coefficients, all *P* < 0.001, and the path analysis results are shown in Table 3.

**Table 3:** Mediation path coefficient analysis.

**Outcome variable**	**Predictive variable**	**β**	**SE**	***t***	***P***	**Bootstrap 95%CI**
HEC	MD	−0.358	0.079	−4.576	< 0.001	(−0.486, −0.209)
MR	HEC	0.537	0.058	5.814	< 0.001	(0.404, 0.665)
	MD	−0.458	0.052	−5.518	< 0.001	(−0.596, −0.313)
JB	MR	−0.471	0.646	−4.404	< 0.001	(−0.706, −0.292)
	MD	0.265	0.242	4.179	< 0.001	(0.104, 0.385)
	HEC	−0.451	0.292	−5.835	< 0.001	(−0.570, −0.286)

MD, moral distress; HEC, hospital ethical climate; MR, moral resilience; JB, job burnout.

The results of the Bootstrap method show that the indirect effect of MD on JB is valid, the total indirect effect is 0.467, and the specific mediating and chain mediating effects are valid. See Table 4. Indirect effect 1: HEC has a mediating effect between MD and JB, and the mediating effect value is 0.358 × 0.451 = 0.161, accounting for 34.48% of the indirect effect. Indirect effect 2: MR has a mediating effect between MD and JB, and the mediating effect value is 0.458 × 0.471 = 0.216, accounting for 46.25% of the indirect effect; Indirect effect 3: HEC and MR play chain mediating roles between MD and JB, and the chain mediating effect value is 0.358 × 0.537 × 0.471 = 0.090, accounting for 19.27% of the total indirect effect.

**Table 4:** Bootstrap analysis of the mediating model.

**Effect**	**Path**	**β**	**The size of effect**	**Boot SE**	**95%confidence interval**	
					**Lower**	**Upper**
Total	MD → JB	0.733	–	0.046	0.638	0.820
Direct	MD → JB	0.265	36.15%	0.071	0.114	0.391
Indirect 1	MD → HEC → JB	0.161	21.96%	0.04	0.091	0.243
Indirect 2	MD → MR → JB	0.216	29.47%	0.065	0.123	0.377
Indirect 3	MD → HEC → MR → JB	0.090	12.28%	0.031	0.047	0.161
	Total Indirect	0.467	63.71%	0.073	0.339	0.629

MD, moral distress; HEC, hospital ethical climate; MR, moral resilience; JB, job burnout.

## 4 Discussion

This study examines the complex interplay between MD, HEC, MR, JB among emergency nurses. While prior research has acknowledged the association between MD and JB, our findings extend this understanding by uncovering a sequential causal pathway through which MD impacts burnout: first by eroding nurses' perception of a supportive HEC (external resource loss), then diminishing their MR (internal resource loss), and ultimately exacerbating JB. This chain-mediating mechanism, validated through structural equation modeling, represents a novel contribution to the literature. By integrating COR theory, we demonstrate how external (HEC) and internal (MR) resources interact dynamically to buffer the impact of MD. These insights not only deepen the theoretical understanding of burnout etiology but also highlight actionable intervention points—such as strengthening ethical climates and moral resilience training—to mitigate burnout in high-stress clinical settings.

### 4.1 Mediating roles of HEC and MR

The results reveal that both HEC and MR significantly mediate the relationship between MD and JB, accounting for 21.96% and 29.47% of the total effect, respectively. Notably, these mediating pathways are empirically supported by significant pairwise correlations: MD showed strong negative associations with both HEC and MR, while HEC and MR each demonstrated inverse correlations with JB. This suggests that MD not only directly contributes to JB but also indirectly exacerbates it by depleting nurses' perception of a supportive HEC and their capacity for MR. However, HEC is not merely a passive target of depletion; it also serves as a critical external resource that buffers the impact of MD. When nurses perceive a positive ethical climate—characterized by supportive relationships with peers, patients, physicians, and managers—they experience reduced psychological pressure and are better equipped to cope with ethical challenges (36). A strong HEC also enhances nurses' professional values and satisfaction, reducing confusion and distress when faced with moral distress (37). Similarly, MR acts as an internal resource that mediates the relationship between MD and JB. Nurses with higher MR are better able to maintain professional integrity, assess patient needs comprehensively, and make ethically sound decisions under pressure (38). This capacity not only reduces the negative impact of MD but also fosters a sense of accomplishment and purpose, further protecting against JB (39).

### 4.2 Chain-mediating effect of HEC and MR

This study further validates the chain-mediating effect of HEC and MR, which accounts for 12.28% of the total effect. Importantly, the observed mediation pathway is reinforced by a significant positive correlation between HEC and MR, this resource synergy reduces the risk of JB, where supportive ethical environments enhance nurses' capacity to cultivate and sustain moral resilience. In environments with strong ethical leadership and a supportive culture, nurses are more likely to access and utilize resources effectively, enhancing their psychological energy and moral resilience (36). This enables them to face moral challenges with a positive attitude, recover more quickly from negative emotions, and ultimately reduce JB (19, 40). These findings extend COR theory by demonstrating how external resources (e.g., ethical climate) and internal resources (e.g., moral resilience) interact sequentially to mitigate the impact of MD. They also highlight the importance of creating organizational conditions that support the development of individual resilience, thereby breaking the cycle of resource depletion and JB.

### 4.3 Practical implications

This study highlights the importance of investing in both external (HEC) and internal (MR) resources to reduce JB caused by MD among emergency nurses. To enhance HEC, ethics training programs and workshops should be implemented to promote ethical decision-making and leadership (41). Establishing ethics clinics can provide valuable guidance and support for nurses facing moral distress (42), while developing a penalty-free reporting system can encourage staff to voice ethical concerns without fear of retribution (43). Strengthening MR is equally important. Psychological training, such as mindfulness and self-reflection exercises, can help nurses process MD and build MR (44). Peer support networks can foster a sense of community and shared responsibility, essential for moral well-being (44). These measures aim to create a supportive and ethical work environment, thereby reducing the impact of moral distress on job burnout among emergency nurses, which can further lead to reduced turnover intentions and improved patient care outcomes. This approach ultimately enhances overall healthcare quality.

## 5 Limitations and future research

Despite its contributions, this study has several limitations. First, the use of convenience sampling may limit the generalizability of the findings. Second, the cross-sectional design restricts our ability to infer causal relationships or observe changes over time. Third, there may be errors in the measurement of variables in this study. For example, in measuring MD, some items of the existing scale fail to fully consider the specific situation of emergency nurses, which may lead to the low MD score of emergency nurses measured in this study (9). Given this, future studies should strive to expand the sample size and increase the sample diversity to improve the generalization of research results. Employing longitudinal designs to explore the long-term effects of MD, HEC, and MR. Developing a MD scale tailored to the specific challenges faced by emergency nurses, thereby improving the accuracy and validity of future studies.

## 6 Conclusions

This study shows a significant positive correlation between MD faced by emergency nurses and JB. Moral distress directly affects nurses' JB and indirectly affects JB through nurses' perceived ethical atmosphere and MR in hospitals. The study further found that HEC and MR play a partial mediating role between MD and JB, and the two together constitute a chain mediating effect. This finding emphasizes the importance of improving the HEC and MR level of clinical nurses, which can not only effectively reduce the impact of MD on nurses' JB but also have important practical significance for improving nursing education and career development, promoting the construction of ethical environment within healthcare institutions, and promoting patient safety and satisfaction. Therefore, this study provides a theoretical basis for healthcare policymakers to improve nurses' working environment and occupational health.

## Data Availability

The raw data supporting the conclusions of this article will be made available by the authors, without undue reservation.
