# Supplementary material for: The effect of moral distress on emergency nurses' job burnout: the mediating roles of hospital ethical climate and moral resilience
Source: Front Public Health. 2025 Mar 5;13:1562209. doi: 10.3389/fpubh.2025.1562209 (PMC11920164; doi:10.3389/fpubh.2025.1562209)
Supplement: Supplementary file 1 [file Table_1.docx]

Table S1 Correlations for variables measured in this study (n = 323)

| Variable | Mean±SD | a | b | c | d | e | f | g | h | i | j |
| --- | --- | --- | --- | --- | --- | --- | --- | --- | --- | --- | --- |
| a. MD | 51.05±15.86 | 1 |  |  |  |  |  |  |  |  |  |
| b. MD1 | 19.72±6.83 | 0.741** | 1 |  |  |  |  |  |  |  |  |
| c. MD2 | 13.54±4.58 | 0.649** | 0.306** | 1 |  |  |  |  |  |  |  |
| d. MD3 | 14.14±6.79 | 0.782** | 0.295** | 0.359** | 1 |  |  |  |  |  |  |
| e. MD4 | 3.64±3.31 | 0.762** | 0.461** | 0.357** | 0.589** | 1 |  |  |  |  |  |
| f. HEC | 90.64±13.83 | -0.274** | -0.214** | -0.132* | -0.231** | -0.217** | 1 |  |  |  |  |
| g. Nurse | 14.57±2.99 | -0.259** | -0.172** | -0.092 | -0.263** | -0.217** | 0.722** | 1 |  |  |  |
| h. Patient | 14.89±2.68 | -0.147** | -0.178** | -0.017 | -0.101 | -0.108 | 0.671** | 0.431** | 1 |  |  |
| i. Doctor | 17.74±4.20 | -0.244** | -0.169** | -0.133* | -0.203** | -0.222** | 0.748** | 0.442** | 0.410** | 1 |  |
| j. Manager | 20.97±4.80 | -0.189** | -0.137* | -0.094 | -0.176** | -0.129* | 0.748** | 0.450** | 0.399** | 0.392** | 1 |
| k. Hospital | 22.48±4.32 | -0.160** | -0.139* | -0.114* | -0.1 | -0.116* | 0.726** | 0.421** | 0.387** | 0.426** | 0.341** |
| l. MR | 40.14±8.14 | -0.481** | -0.380** | -0.286** | -0.372** | -0.361** | 0.488** | 0.422** | 0.379** | 0.347** | 0.333** |
| m. MR1 | 9.59±2.84 | -0.403** | -0.320** | -0.261** | -0.299** | -0.297** | 0.345** | 0.288** | 0.311** | 0.230** | 0.255** |
| n. MR2 | 12.51±3.36 | -0.329** | -0.271** | -0.175** | -0.257** | -0.245** | 0.335** | 0.279** | 0.281** | 0.232** | 0.183** |
| o. MR3 | 10.39±3.07 | -0.295** | -0.218** | -0.147** | -0.250** | -0.247** | 0.335** | 0.321** | 0.212** | 0.242** | 0.237** |
| p. MR4 | 7.65±2.19 | -0.347** | -0.275** | -0.250** | -0.251** | -0.232** | 0.383** | 0.316** | 0.278** | 0.296** | 0.295** |
| q. JB | 61.86±24.19 | 0.612** | 0.444** | 0.420** | 0.469** | 0.472** | -0.714** | -0.563** | -0.518** | -0.551** | -0.530** |
| r. EE | 25.62±10.94 | 0.573** | 0.419** | 0.366** | 0.440** | 0.471** | -0.668** | -0.524** | -0.477** | -0.540** | -0.481** |
| s. DP | 14.53±6.20 | 0.510** | 0.374** | 0.373** | 0.380** | 0.374** | -0.625** | -0.483** | -0.447** | -0.497** | -0.470** |
| t. rPA | 21.71±9.85 | 0.546** | 0.391** | 0.390** | 0.424** | 0.401** | -0.617** | -0.496** | -0.461** | -0.440** | -0.472** |

| Variable | Mean±SD | k | l | m | n | o | p | q | r | s | t |
| --- | --- | --- | --- | --- | --- | --- | --- | --- | --- | --- | --- |
| a. MD | 51.05±15.86 |  |  |  |  |  |  |  |  |  |  |
| b. MD1 | 19.72±6.83 |  |  |  |  |  |  |  |  |  |  |
| c. MD2 | 13.54±4.58 |  |  |  |  |  |  |  |  |  |  |
| d. MD3 | 14.14±6.79 |  |  |  |  |  |  |  |  |  |  |
| e. MD4 | 3.64±3.31 |  |  |  |  |  |  |  |  |  |  |
| f. HEC | 90.64±13.83 |  |  |  |  |  |  |  |  |  |  |
| g. Peers | 14.57±2.99 |  |  |  |  |  |  |  |  |  |  |
| h. Patients | 14.89±2.68 |  |  |  |  |  |  |  |  |  |  |
| i. Physicians | 17.74±4.20 |  |  |  |  |  |  |  |  |  |  |
| j. Managers | 20.97±4.80 |  |  |  |  |  |  |  |  |  |  |
| k. Hospitals | 22.48±4.32 | 1 |  |  |  |  |  |  |  |  |  |
| l. MR | 40.14±8.14 | 0.327** | 1 |  |  |  |  |  |  |  |  |
| m. MR1 | 9.59±2.84 | 0.206** | 0.678** | 1 |  |  |  |  |  |  |  |
| n. MR2 | 12.51±3.36 | 0.277** | 0.762** | 0.322** | 1 |  |  |  |  |  |  |
| o. MR3 | 10.39±3.07 | 0.218** | 0.714** | 0.286** | 0.355** | 1 |  |  |  |  |  |
| p. MR4 | 7.65±2.19 | 0.218** | 0.668** | 0.329** | 0.383** | 0.335** | 1 |  |  |  |  |
| q. JB | 61.86±24.19 | -0.448** | -0.728** | -0.499** | -0.493** | -0.527** | -0.562** | 1 |  |  |  |
| r. EE | 25.62±10.94 | -0.420** | -0.654** | -0.418** | -0.459** | -0.488** | -0.499** | 0.920** | 1 |  |  |
| s. DP | 14.53±6.20 | -0.382** | -0.615** | -0.470** | -0.380** | -0.422** | -0.501** | 0.849** | 0.705** | 1 |  |
| t. rPA | 21.71±9.85 | -0.394** | -0.675** | -0.466** | -0.463** | -0.488** | -0.511** | 0.900** | 0.707** | 0.675** | 1 |

MD, moral distress; MD1, individual responsibility; MD2, not in the patient's best interest; MD3, value conflict; MD4, harming patient's interest; HEC, hospital ethical climate; MR, moral resilience; MR1, responses to moral adversity; MR2, moral efficacy; MR3, relational integrity; MR4, personal integrity; JB, job burnout; EE, emotional exhaustion; DP, depersonalization; rPA, reduced personal accomplishment. **Statistical significance at the level of 0.01 (two-tailed). SD, standard deviation
